# Chinese adult segmentation according to health skills and analysis of their use for smart home: a cross-sectional national survey

**DOI:** 10.1186/s12913-022-08126-8

**Published:** 2022-06-10

**Authors:** Feiying He, Yibo Wu, Jiao Yang, Keer Chen, Jingyu Xie, Yusupujiang Tuersun, Lehuan Li, Fangjing Wu, Yifan Kan, Yuqian Deng, Liping Zhao, Jingxi Chen, Xinying Sun, Shengwu Liao, JiangYun Chen

**Affiliations:** 1 Southern Medical University, No.1023-1063 Shatai Road, Baiyun District, Guangzhou City, Guangdong Province China; 2 School of Public Health, Peking University, No.38 Xueyuan Road, Haidian District, Beijing City, China; 3 School of Health Management, Southern Medical University, No.1023-1063 Shatai Road, Baiyun District, Guangzhou City, Guangdong Province China; 4 School of Public Health, Southern Medical University, No.1023-1063 Shatai Road, Baiyun District, Guangzhou City, Guangdong Province China; 5 Xiangya School of Nursing, Central South University, No. 172 Tongzipo Road, Yuelu District, Changsha City, Hunan Province China; 6 The Second Xiangya Hospital, Central South University, No.139 Renmin Road, Changsha City, Hunan Province China; 7 School of Languages and Communication Studies of Beijing Jiaotong University, No.3 Shangyuancun, Haidian District, Beijing City, China; 8 Department of Health Management, Southern Hospital of Southern Medical University, 1838 Guangzhou Avenue North, Baiyun District, Guangzhou, Guangdong China; 9 Institute of Health Management, Southern Medical University, No.1023-1063 Shatai Road, Baiyun District, Guangzhou City, Guangdong Province China

**Keywords:** Smart Home, Digital Health, Health skills, Clustering, China Family Health Index Survey

## Abstract

**Background:**

Digital health has become a heated topic today and smart homes have received much attention as an important area of digital health. Smart home is a device that enables automation and remote control in a home environment via the internet. However, most of the existing studies have focused on discussing the impact of smart home on people. Only few studies have focused on relationship between health skills and use of smart home.

**Aims:**

To analyze the health skills of Chinese adults and segment them to compare and analyze the use of smart home for each group.

**Methods:**

We used data from 11,031 participants aged 18 and above. The population was clustered based on five health skills factors: perceived social support, family health, health literacy, media use, and chronic diseases self-behavioral management. A total of 23 smart homes were categorized into three sub-categories based on their functions: entertainment smart home, functional smart home, and health smart home. We analyzed demographic characteristics and utilization rate of smart home across different cluster. Each groups’ features and the differences in their needs for smart home functions were compared and analyzed.

**Results:**

As a result of the survey on health skills, three groups with different characteristics were clustered: good health skills, middle health skills, and poor health skills. The utilization rate of smart home was the highest was good health skills group (total smart home: 92.7%; entertainment smart home: 61.1%, functional smart home: 77.4%, and health smart home: 75.3%; *P* < 0.001). For entertainment smart home, smart TV had the highest utilization rate (good health skills: 45.7%; middle health skills: 43.5%, poor health skills: 33.4%, *P *< 0.001). For functional smart home, smart washing machine (good health skills: 37.7%, middle health skills: 35.11%, poor health skills: 26.5%; *P *< 0.001) and smart air conditioner (good health skills: 36.0%, middle health skills: 29.1%, poor health skills: 24.6%) were higher than other of this category. For health smart home, sports bracelet has the highest utilization rate (good health skills: 37.3%, middle health skills: 24.5%, poor health skills: 22.8%).

**Conclusion:**

People can be divided into different categories based on health skill profiles, those with good health skills had a better utilization rate of smart home. The government and smart home companies need to focus on people with poor smart home use in various ways to promote their use of smart homes for personal health management.

**Supplementary Information:**

The online version contains supplementary material available at 10.1186/s12913-022-08126-8.

## Introduction

Along with accelerated industrialization, urbanization, and population aging, China's disease spectrum continues to change. The death rate from chronic non-communicable diseases is in the proportion to 88% of all deaths. The resulting disease burden accounts for over 70% of the total disease burden. Chinese government developed a "Healthy China" strategy in 2017 to improve the health literacy of residents, prevent diseases, and improve the quality of life of residents [1]. The Internet of Things (IoT) has experienced rapid growth in the past decade, covering many fields, especially digital health.

Following the development trend of technology, the World Health Organization (WHO) has proposed a global digital health strategy for 2020–2025, advocating the promotion of digital health and the application of digital health to achieve the goal of universal health [2]. To help drive the development of health in China, the government is also using Internet technology. It has been continuously focusing on the innovative home sector since 2008, promulgating policy documents to support the development of smart homes [3]. The report "China Internet Development 2021" released by the Internet Society of China shows that China's Internet penetration rate has reached 70.4%, the internet of things market size reached 1.7 trillion yuan, the artificial intelligence market size rose to 303.1 billion yuan, and the market size of online medical and health services climbed to 196.1 billion yuan [4], which in general means that China has a good Internet foundation to carry out digital health.

Smart home means the growing variety of devices and appliances in a home environment that are connected to each other via the internet for automatic and remote control to enhance the home life experience [5]. Previous studies have shown that smart home play an important role in digital health and that the use of smart homes can improve human health outcomes. Smart home can relieve stress [6], alleviate loneliness [7], increase security [8], enhance ease of movement [9], improve quality of life [10], and improve blood pressure [11]. These benefits are particularly evident for special groups. Firstly, smart home can help people with disabilities who have communication difficulties to enhance their understanding of speech and improve their communication [12]. Secondly, remote support services can help enhance the sense of security provided by people with intellectual and related developmental disabilities [13]. In addition, smart home can improve personal safety and social communication issues for older people [14].

Health skills is defined as the ability to assess, identify, and target aspects of health in various settings in order to improve the overall wellbeing of an individual or organization [15]. Through a review of the literature, we identified five health skills that influence health management. Firstly, high social support is associated with better health-related quality of life [16]. Secondly, family functioning has also been shown to be strongly associated with health-related quality of life [17]. In addition, social media is an effective tool to promote COVID-19 prevention behaviors among the general public, to increase understanding of disease and health, and to better prevent disease. In addition, chronic diseases are important determinants of health-related quality of life [18]. Healthy habits and behaviors, such as a regular routine and exercise in leisure time, can improve health outcomes [19]. Finally, a survey showed that health literacy was a protective factor for depression during the COVID-19 pandemic [20]. Thus, five important aspects related to health skills can be identified as: social support, family health, media use, self-management, and health literacy. These aspects related to health skills can help people develop healthy behaviors [15].

### The present study

In summary, many of the literature reveals that smart home can effectively contribute to improved health outcomes in the context of the eHealth. Health skills can help people manage their own health. But few studies concerned about people's health skills and whether there is variability in smart home use between users with different health skill profiles. Promoting the use of smart homes is important for the implementation of digital health strategies and health promotion. Therefore, there is a need to understand the health skills of Chinese adults and segment them to compare and analyze the use of smart home for each group. This study is a large sample population survey conducted in China with the aim of finding adult segmentation based on health skills for smart home use, and identifying the utilization rate of smart home of each group in regards to smart home categories.

## Methods

### Data and procedure

The data used in this study is conducted in 23 provinces, 5 autonomous regions, and 4 municipalities directly under the central government from July to September 2021. The survey is a multi-stage sampling, using the random number table method to select 2–6 cities from each noncapital prefecture-level administrative region of each province and an autonomous region, a total of 120 cities; based on the data results of "the seventh national census in 2021", quota sampling (quota attributes are gender, age, and urban–rural distribution) was conducted for 120 cities, so that the gender, age and urban–rural distribution of the samples basically conform to the demographic characteristics. At least one surveyor or a panel of surveyors was recruited in each city, with each surveyor responsible for collecting 30–90 questionnaires and each panel responsible for collecting 100–200 questionnaires. The surveyor uses the Online Questionnaire Star platform (https://www.wjx.cn/) to distribute the questionnaire to each person, obtain informed consent from each participating respondent and record the questionnaire number issued to that person. Subjects were included in the study if they were ≥ 18 years old, provided written informed consent and volunteered to participate in the study. Finally, 11,031 valid questionnaires were obtained that have high quality and accurate national representation and comply with the ethical review rules (JNUKY-2021–018).

### Variables

#### Characteristic variable

The characteristic variable in this study included respondents' socio-economic background (age, gender, income, Hukou, residence, education, public insurance, location recently, chronic disease, Disability, work status and Politics), family characteristics (Marriage, family type, number of children, household) and lifestyle (drinking status). See Supplementary Table S1 for details of definitions and classifications.

#### Smart home use

Through a literature review, expert consultation and a search of the most widely used shopping sites in China, Taobao etc., we identified 23 types of smart home that are currently in widespread use at home and abroad. Then, we divided them into three categories according to the functions of smart home: entertainment SHU (consists of smart TV, VR glasses, body sensing car, smart speaker), function assistance SHU (consists of a smart robot, smart lighting, smart washing machine, smart switch, smart door lock, smart toilet, smart mosquito repellent, electric curtain, smart air conditioner, smart clothes hanger, smart monitoring) and health SHU (consists of sports bracelet, temperature and humidity sensor, smart socket, danger button, smoke transducer, body fat scale, air purifier, smart medicine cabinet).

### The utilization breadth of smart home

Each respondent will be asked to check off our list of the 23 most common smart homes in China. The utilization breadth of smart home is generated by the following equation: $utilization\;breadth=\:{\mathrm n}_{\mathrm s}/{\mathrm N}_{\mathrm s}\ast100\%$ (n_s_ refers to number of smart home checked in the list, N_s_ refers to the total number of smart homes).

### The utilization rate of smart home

The utilization rate of smart home is generated by the following equation: $utilization\;rate=\:{\mathrm n}_{\mathrm p}/{\mathrm N}_{\mathrm p}\ast100\%\;$  (n_p_ refers to number of person who reported uses the smart home, N_p_ refers to the total number of people surveyed).

#### Health skill

We have identified five important aspects related to health skills: social support, family health, media use, self-management, and health literacy. The five aspects related to health skills were measured using the following tools.

Social support was measured using the Perceived Social Support Scale (PSSS) based on the Zimet Perceived Social Support Scale. A 12-item scale divided into three dimensions: family support, friend support, and other supports, as shown in Table S2 in the attached table. "strongly disagree", "slightly disagree", "neutral", "slightly agree", "agree", "Strongly agree" seven options, these seven options are assigned a score of 1–7 (Strongly disagree = 1). The higher the score, the higher the perceived social support. The alpha coefficients for family support, friend support, other supports and the full scale were 0.87, 0.85, 0.91 and 0.88 for the sample of 275 cases (139 males and 136 females) respectively, with retest reliability of 0.85, 0.75, 0.72 and 0.85 [21].

Family health was measured using the Family Health Scale-Short Form (FHS-SF) based on the AliceAnn Crandall [22] (Supplementary Table S3). For each item, subjects rated "strongly disagree", "somewhat disagree", "neither agree nor disagree", "somewhat agree " and "strongly agree", with the three dimensions (7 items) other than "family health resources" being assigned a value of 1–5 in order ("strongly disagree" = 1), while the three items for "family health resources" were assigned the opposite value ("strongly agree" = 1). Cronbach's alpha for the 10-item scale was 0.80 and Cronbach's alpha for the FHS-SF was 0.84.

Media use was measured using a 5-point Likert scale with seven items. The scale has five options: "never use", "occasionally use", "sometimes use", "often use" and "almost every day" that are assigned a value of 0–4 in order (never use = 0), see Table S4 in the Supplementary Material.

Self-management was measured using the Chronic Disease Self-management Study Measure (CDSMS) developed by Lorig [23, 24]. The scale is divided into two sub scales: self-management behavior and self-management effectiveness. The self-management behavior scale consists of 6 items including exercise, cognitive symptom management practices, and communication with a doctor. The six items were rated on a scale of 0–4 (not done = 0), with higher scores resulting in higher status. See Table S5 in the Supplementary Material for details. The Cronbach coefficient for the CDSMS was 0.72–0.75.

Health literacy was measured using the Short-Form Health Literacy Instrument (HLS-SF12) developed by Tuyen V Duong [25]. The scale has 12 items covering the three health domains of health care, disease prevention, and a health promotion, as detailed in Table S6 in the Supplementary Material. “very difficult”, “difficult”, " easy", and "very easy" for each item, in order of assignment from 1 to 4 ("very difficult" = 1). The higher the score is, the higher the health literacy is. This scale has high reliability with a Cronbach's alpha of 0.85.

### Statistical analysis

Firstly, Non-hierarchical K-means cluster analysis was conducted to construct segmentation based on five aspects related to health skills: social support, family health, media use, self-management, and health literacy. Suppose a factor has a relatively large cluster center value. In that case, it can be characterized as a cluster that is positively affected by the factor. Then we conducted the analysis of variance (ANOVA) to explore the differences of the health skills between the cluster groups. After that, we described the demographic characteristics in the three clusters. The demographic characteristics of each group were compared via chi-square test. Secondly,

analysis of variance (ANOVA) was conducted to explore the distribution of utilization breadth between the cluster and demographic characteristics groups. The chi-square test was used to compare the distribution of utilization rate between different cluster and demographic characteristics groups. Thirdly, subgroup analysis of utilization rate of smart home was performed, included residence (urban/rural), gender (female/male), and age (45 ~ 59/60 ~ 75/76 ~). A two-sided test (α < 0.05) was employed to determine statistical significance. Stata version 16 (2017, College Station, Texas 77,845 USA) were used for statistical analysis.

## Results

### Segmentation based on health skills

As the clustering was more balanced in each group when clustered into three groups and there were significant differences in health skills characteristics among the groups, the clustering can be judged that is convincing when clustered into three groups. Table 1 showed the results of the clustering defined into three groups. The analysis shown that there was a significant difference among three clusters in Chronic self-behavior management, Health literacy, Media use, Perceive social support and Family health (*p* < 0.05).

**Table 1:** Descriptive analysis of five health skill among different cluster (mean ± SD)

**Categories/Definition**	Cluster 1 (*n* = 2679)	Cluster 2 (*n* = 4589)	Cluster 3 (*n* = 3763)	*p*-value
	Good Health Skills	Middle Health Skills	Poor Health Skills	
PSSS	68.42 ± 10.54	65.46 ± 9.07	47.99 ± 9.33	< 0.001
**FLS-SF**	40.96 ± 5.94	41.44 ± 4.71	31.68 ± 4.06	< 0.001
FLS_ social and emotional health processes	13.26 ± 1.82	13.12 ± 1.65	9.41 ± 2.04	< 0.001
FLS_ healthy lifestyle	8.88 ± 1.25	8.83 ± 1.13	6.36 ± 1.43	< 0.001
FLS_ health resources	10.32 ± 3.96	11.25 ± 2.99	9.65 ± 2.00	< 0.001
FLS_ external social supports	8.50 ± 1.37	8.23 ± 1.30	6.26 ± 1.35	< 0.001
Media use	17.19 ± 4.37	10.50 ± 3.65	11.12 ± 4.47	< 0.001
CDSMS	18.82 ± 5.30	10.45 ± 2.93	13.18 ± 5.08	< 0.001
HLS-SF12	40.99 ± 5.38	36.94 ± 4.94	33.36 ± 5.69	< 0.001

*PSSS* Perceived Social Support Scale, *FLS-SF* Family Health Scale-Short Form, *CDSMS* Chronic Disease Self-management Study Measure, *HLS-SF12* Short-Form Health Literacy Instrument

**Fig. 1:**
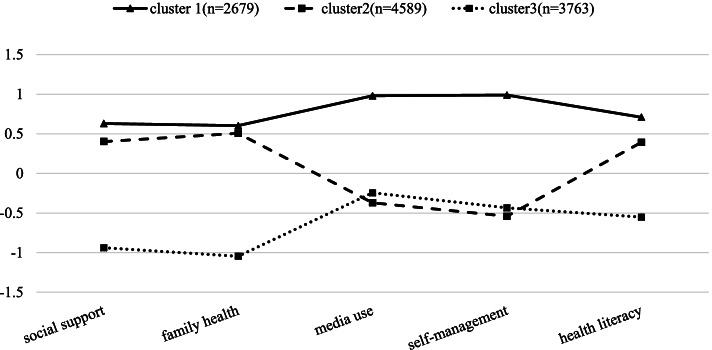
Clusters for health skills related factor of Chinese adults. Note: The definition of the classes: class 1, good health skills; class 2, middle health skills; class 3, poor health skills. Self-management: chronic disease self-behavioral management

The clusters were defined and named based on the level of health skills and characteristics. cluster 1 ‘good health skills’ is a group that the mean values of each variable were each higher than their overall respective mean values. they take the best health skills. cluster 2 ‘middle health skills’ is a group whose mean values are above average except for media use and chronic self-behavior management. cluster 3 ‘poor health skills’ is the poorest health skills group that means values lower than average. The difference among the factors for each group can be seen in Fig. 1.

### Descriptive statistics and correlations

Table 2 showed the demographic and covariant characteristics of each cluster. The proportion of females was higher in all groups. And the proportion of young and middle-aged people aged 19 ~ 45 was higher, and the proportion of people aged over 91 was lower in all groups (*P* < 0.001). There was a significant difference in some characteristic variables among the three groups. This is mainly in terms of income, education and work status (*P* < 0.001). Cluster 1 has a significantly higher proportion of people earning 7501 or more than cluster 2 and cluster 3. In addition, cluster 1 has a higher number of college and bachelor degrees and above than the other two clusters. Besides, cluster 2 and cluster 3 have a significantly higher proportion of no fixed occupation than cluster 1.

**Table 2:** Demographic characteristics of each cluster [n (%)]

Categories/Definition		Cluster 1 (*n* = 2679)	Cluster 2 (*n* = 4589)	Cluster 3 (*n* = 3763)	*P*-value
		Good health Skills	Middle Health Skills	Poor Health Skills	
**Gender**	Man	1330 (49.6)	1846 (40.2)	1857 (49.3)	< 0.001
	Woman	1349 (50.4)	2743 (59.8)	1906 (50.7)	
**Age**	~ 18	280 (10.5)	433 (9.4)	352 (9.4)	< 0.001
	19 ~ 45	1766 (65.9)	2532 (55.2)	2303 (61.2)	
	46 ~ 59	502 (18.7)	1025 (22.3)	691 (18.4)	
	60 ~ 75	110 (4.1)	445 (9.7)	320 (8.5)	
	76 ~ 90	17 (0.6)	150 (3.3)	93 (2.5)	
	91 ~	4 (0.1)	4 (0.1)	4 (0.1)	
**Income**	~ 3000	532 (19.9)	1380 (30.1)	1334 (35.5)	< 0.001
	3001 ~ 7500	1299 (48.5)	2294 (50.0)	1732 (46.0)	
	7501 ~	848 (31.7)	915 (19.9)	697 (18.5)	
**Hukou**	Urban	1787 (66.7)	2567 (55.9)	2006 (53.3)	< 0.001
	Rural	892 (33.3)	2022 (44.1)	1757 (46.7)	
**Education**	Illiteracy	26 (1.0)	163 (3.6)	189 (5.0)	< 0.001
	Below secondary school	272 (10.2)	1139 (24.8)	777 (20.6)	
	Secondary Education	424 (15.8)	847 (18.5)	707 (18.8)	
	College and Bachelor	1658 (61.9)	2201 (48.0)	1891 (50.3)	
	Master and PhD	299 (11.2)	239 (5.2)	199 (5.3)	
**Work status**	Working	1297 (48.4)	1953 (42.6)	1387 (36.9)	< 0.001
	Retired	161 (6.0)	438 (9.5)	285 (7.6)	
	Student	933 (34.8)	1217 (26.5)	1164 (30.9)	
	No fixed occupation	288 (10.8)	981 (21.4)	927 (24.6)	
**Family type**	Nuclear family	1662 (62.0)	2847 (62.0)	2038 (54.2)	< 0.001
	Conjugal family	429 (16.0)	625 (13.6)	709 (18.8)	
	Backbone family	251 (9.4)	635 (13.8)	459 (12.2)	
	Single-parent family	75 (2.8)	168 (3.7)	175 (4.7)	
	Other	262 (9.8)	314 (6.8)	382 (10.2)	
**Location**	Eastern region	1427 (53.3)	2306 (50.3)	1877 (49.9)	0.075
	Central region	658 (24.6)	1196 (26.1)	998 (26.5)	
	Western region	593 (22.1)	1087 (23.7)	886 (23.6)	
**Residence**	Urban	2134 (79.7)	3271 (71.3)	2603 (69.2)	< 0.001
	Rural	545 (20.3)	1318 (28.7)	1160 (30.8)	
**Marriage**	Unmarried	1205 (45.0)	1582 (34.5)	1576 (41.9)	< 0.001
	Married	1421 (53.0)	2803 (61.1)	2002 (53.2)	
	Divorce	43 (1.6)	66 (1.4)	98 (2.6)	
	Widowed	10 (0.4)	138 (3.0)	87 (2.3)	
**Children**	No	1415 (52.8)	1819 (39.6)	1828 (48.6)	< 0.001
	One	772 (28.8)	1350 (29.4)	937 (24.9)	
	Two	418 (15.6)	1064 (23.2)	752 (20.0)	
	Three or more	74 (2.8)	356 (7.8)	246 (6.5)	
**Household**	No	267 (10.0)	301 (6.6)	495 (13.2)	< 0.001
	One	810 (30.3)	1479 (32.3)	1327 (35.3)	
	Two	791 (29.6)	1389 (30.3)	963 (25.6)	
	Three	440 (16.4)	739 (16.1)	507 (13.5)	
	Four	150 (5.6)	323 (7.0)	214 (5.7)	
	Five or more	217 (8.1)	355 (7.7)	254 (6.8)	
**Public insurance**	No	833 (31.1)	1325 (28.9)	779 (20.7)	< 0.001
	Yes	1846(68.9)	3264(71.1)	2984(79.3)	
**Chronic disease**	No	2352 (87.8)	3595 (78.3)	3037 (80.7)	< 0.001
	One	242 (9.0)	661 (14.4)	479 (12.7)	
	Two or more	85(3.2)	333(7.3)	247(6.6)	
**Disability**	No	2603 (97.2)	4464 (97.3)	3617 (96.1)	0.001
	Yes	76 (2.8)	125 (2.7)	146 (3.9)	
**Drinking status**	Yes, within 30 days	1491 (55.7)	2842 (61.9)	2245 (59.7)	< 0.001
	Yes, before 30 days	342 (12.8)	510 (11.1)	456 (12.1)	
	No	846 (31.6)	1237 (27.0)	1062 (28.2)	

As shown in Table 3, the ANOVA analysis results show that there are significant differences in each group in the utilization breadth of total smart home, entertainment smart home, functional smart home, and health smart home (*P* < 0.001). Cluster 1 had a higher breadth use of smart home in total and different categories than other groups. Cluster 2 was slightly higher than cluster 3 in terms of the breadth utilization of the entertainment smart home and the functional smart home. While, cluster 3 is slightly higher than cluster 2 in terms of the breadth utilization of the health smart home. Table 3 also described the utilization breadth of smart home across demographics and characteristic variables. There was a higher utilization rate of total smart home in respondents were female, having higher income and education, urban residence, living in a nuclear family, covered by public insurance, without chronic disease or disability (*P* < 0.001). However, in the entertainment smart home category, the utilization breadth of smart home by females is lower than the use of smart homes by males.

**Table 3:** Cluster and demographic differences by utilization breadth of smart home (%)

Categories/Definition		Total smart home		Entertainment smart home		Functional smart home		Health smart home	
		Utilization breadth	*P*-value	Utilization breadth	*P*-value	Utilization breadth	*P*-value	Utilization breadth	*P*-value
**Cluster**	Cluster 1 (Good health skills)	20.5	< 0.001	22.2	< 0.001	20.0	< 0.001	20.3	< 0.001
	Cluster 2 (Middle health skills)	13.0		15.9		12.6		12.2	
	Cluster 3 (Poor health skills)	13.5		15.1		13.5		12.7	
**Gender**	Man	14.9	0.403	17.7	0.043	14.3	0.131	14.2	0.079
	Woman	15.1		16.7		15.0		14.4	
**Age**	~ 18	14.9	< 0.001	17.9	< 0.001	14.2	< 0.001	14.3	< 0.001
	19 ~ 45	16.2		18.4		15.7		15.7	
	46 ~ 59	13.8		15.7		13.9		12.8	
	60 ~ 75	11.1		12.6		11.4		9.8	
	76 ~ 90	8.8		10.4		9.3		7.2	
	91 ~	16.3		10.4		17.4		17.7	
**Income**	~ 3000	11.3	< 0.001	14.3	< 0.001	11.2	< 0.001	10.0	< 0.001
	3001 ~ 7500	14.8		16.9		14.6		14.1	
	7501 ~	20.2		21.5		19.6		20.4	
**Hukou**	Urban	16.5	< 0.001	18.4	< 0.001	16.0	< 0.001	16.4	< 0.001
	Rural	12.9		15.5		13.0		11.4	
**Education**	Illiteracy	9.2	< 0.001	10.8	< 0.001	10.1	< 0.001	7.0	< 0.001
	Below secondary school	11.5		14.6		11.7		9.5	
	Secondary Education	14.6		17.1		14.4		13.6	
	College and Bachelor	16.0		17.7		15.5		15.7	
	Master and PhD	22.0		24.0		20.3		23.3	
**Work status**	In-service	17.0	< 0.001	18.7	< 0.001	16.6	< 0.001	16.6	< 0.001
	Retired	12.7		13.9		12.7		12.0	
	Student	14.7		17.3		14.0		14.4	
	No fixed occupation	12.2		15.0		12.6		10.2	
**Family type**	Nuclear family	15.2	< 0.001	17.6	< 0.001	14.7	0.011	14.6	< 0.001
	Conjugal family	15.5		17.1		15.3		15.1	
	Backbone family	13.9		15.7		14.2		12.6	
	Single-parent family	13.6		15.3		13.6		12.9	
	Other	14.9		16.9		14.9		14.0	
**Location**	Eastern region	15.3	0.074	16.8	< 0.001	14.8	0.849	15.1	< 0.001
	Central region	15.1		17.7		15.0		13.9	
	Western region	14.3		17.3		14.2		13.0	
**Residence**	Urban	18.2	< 0.001	15.8	< 0.001	15.9	< 0.001	16.3	< 0.001
	Rural	14.3		11.9		10.0		11.7	
**Marriage**	Unmarried	15.5	< 0.001	18.1	< 0.001	14.7	< 0.001	15.2	< 0.001
	Married	14.9		16.7		14.8		13.9	
	Divorce	15.3		16.3		15.7		14.4	
	Widowed	9.6		11.3		10.3		7.7	
**Children**	No	15.8	< 0.001	18.2	< 0.001	15.1	< 0.001	15.6	< 0.001
	One	15.3		16.7		15.1		14.9	
	Two	14.0		16.6		14.3		12.3	
	Three or more	10.8		13.5		11.4		8.7	
**Household**	No	16.4	< 0.001	18.1	< 0.001	16.2	0.001	15.9	< 0.001
	One	14.6		16.2		14.7		13.8	
	Two	14.9		17.3		14.3		14.6	
	Three	15.2		17.8		15.1		14.0	
	Four	16.2		19.1		15.9		15.2	
	Five or more	13.7		16.8		12.9		13.3	
**Public insurance**	No	14.2	< 0.001	16.5	0.001	14.0	< 0.001	13.3	< 0.001
	Yes	15.8		17.8		15.5		15.4	
**Chronic disease**	No	15.4	< 0.001	17.6	< 0.001	15.1	< 0.001	14.8	< 0.001
	One	13.0		15.1		12.7		12.4	
	Two or more	13.1		15.0		13.2		12.0	
**Disability**	No	15.1	0.550	17.2	0.648	14.8	0.863	14.4	0.400
	Yes	13.2		15.6		13.1		12.2	
**Drinking status**	Within 30 days	14.5	0.015	16.6	0.026	14.5	0.255	13.5	< 0.001
	Before 30 days	15.4		17.4		15.0		14.8	
	No	15.8		18.2		15.0		15.7	

Table 4 showed that there were significant differences among the three groups in the use of total smart home in three categories. Among them, the good health skills group had a higher utilization rate of smart homes, especially functional homes, with a utilization rate of 77.42%. It was much higher than that in the middle health skills groups and poor health skills. Except that the utilization rate of entertainment smart homes in the middle health skills groups was higher than that in the poor health skills group, the utilization rate of functional and health smart home was lower than that in the poor health skills group. For the entertainment class, smart TV had the highest utilization rate, while VR glasses and body sensing cars had a lower utilization rate. For the functional class, the utilization rates of smart washing machines and smart air conditioners were high, while the utilization rates of electric current, smart clothes changers, smart mosquito reply, and smart robots were low. For health smart home, sports brace, body fat scale, and air purifier were used more frequently, while temperature and humidity sensor, danger button, smoke transmitter, and smart medicine cabinet were used less frequently.

**Table 4:** Cluster difference by the utilization rate of smart home [n (%)]

**Categories/Definition**	Cluster 1 (*n* = 2679)	Cluster 2 (*n* = 4589)	Cluster 3 (*n* = 3763)	*P*-value
	Good health skills	Middle health skills	Poor health skills	
**Total smart home**^a^	2484 (92.7)	3749 (81.7)	3083 (81.9)	< 0.001
**Entertainment smart home**^a^	1636 (61.1)	2364 (51.5)	1725 (45.8)	< 0.001
Smart TV	1225 (45.7)	1997 (43.5)	1256 (33.4)	< 0.001
VR glasses	258 (9.6)	125 (2.7)	252 (6.7)	< 0.001
Body sensing car	220 (8.2)	93 (2.0)	201 (5.3)	< 0.001
Smart speaker	681 (25.4)	706 (15.4)	558 (14.8)	< 0.001
**Functional smart home**^a^	2074 (77.4)	2857 (62.3)	2405 (63.9)	< 0.001
Smart robot	333 (12.4)	241 (5.3)	308 (8.2)	< 0.001
Smart lighting	571 (21.3)	544 (11.9)	537 (14.3)	< 0.001
Smart washing machine	1009 (37.7)	1611 (35.1)	997 (26.5)	< 0.001
Smart switch	545 (20.3)	521 (11.4)	529 (14.1)	< 0.001
Smart door lock	570 (21.3)	551 (12.0)	530 (14.1)	< 0.001
Smart toilet	443 (16.5)	469 (10.2)	379 (10.1)	< 0.001
Smart mosquito repellent	338 (12.6)	239 (5.2)	322 (8.6)	< 0.001
Electric curtain	312 (11.7)	162 (3.5)	292 (7.8)	< 0.001
Smart air conditioner	963 (36.0)	1337 (29.1)	926 (24.6)	< 0.001
Smart clothes hanger	343 (12.8)	293 (6.4)	331 (8.8)	< 0.001
Smart monitoring	417 (15.6)	400 (8.7)	416 (11.1)	< 0.001
**Health smart home**^a^	2018 (75.3)	2531 (55.2)	2115 (56.2)	< 0.001
Sports bracelet	999 (37.3)	1124 (24.5)	859 (22.8)	< 0.001
Temperature and humidity sensor	272 (10.2)	170 (3.7)	261 (6.94)	< 0.001
Smart socket	454 (17.0)	392 (8.5)	455 (12.1)	< 0.001
Danger button	245 (9.2)	131 (2.9)	262 (7.0)	< 0.001
Smoke transducer	261 (9.7)	231 (5.0)	262 (7.0)	< 0.001
Body fat scale	994 (37.1)	1291 (28.1)	853 (22.7)	< 0.001
Air purifier	719 (26.8)	646 (14.1)	520 (13.8)	< 0.001
Smart medicine cabinet	225 (8.4)	104 (2.3)	222 (5.9)	< 0.001

^a^ The utilization rate was calculated any use of smart home

### Subgroup analysis

According to Table 5, smart home use is generally better among urban populations, middle-aged people (45–49 years) than among rural populations, older people (76-years). There were gender differences in the use of VR glasses, body-sensing cars, smart TV, smart brace, smart medicine cabinet, smart socket, temperature, and humidity sensors, and the use of men was higher than that of women. At the same time, gender differences also had differences in the use of smart clothes hangers, which shows that the use of women was higher than that of men.

**Table 5:** Residence, gender, and age subgroup analysis of utilization rate of smart home

Categories/Definition	Residence(%)		*P*-value	Gender(%)		*P*-value	Age(%)			*P*-value
	**Urban**	**Rural**		**Male**	**Female**		**45 ~ 59**	**60 ~ 75**	**76 ~ **	
**Total smart home**^a^	86.9	78.0	< 0.001	83.6	85.2	0.022	82.7	70.9	65.8	< 0.001
**Entertainment smart home**^a^	54.0	46.2	< 0.001	52.4	51.5	0.303	51.0	40.5	34.2	< 0.001
Smart speaker	19.6	12.3	< 0.001	18.1	15.2	0.218	14.4	9.7	8.1	< 0.001
VR glasses	6.6	3.6	< 0.001	7.1	5.9	< 0.001	4.1	2.7	1.8	< 0.001
Body sensing car	5.1	3.5	0.001	5.7	4.8	< 0.001	2.7	3.5	4.4	< 0.001
Smart TV	41.7	37.7	< 0.001	39.7	33.3	0.079	41.8	34.3	27.2	< 0.001
**Functional smart home**^a^	69.2	59.3	< 0.001	65.2	67.6	0.011	66.2	54.6	52.2	< 0.001
Smart robot	9.3	4.7	< 0.001	8.6	7.5	0.031	7.1	6.1	2.9	0.001
Smart air conditioner	30.5	25.8	< 0.001	27.8	30.4	0.003	30.2	24.5	18.0	< 0.001
Smart lighting	15.8	12.8	< 0.001	14.9	15.0	0.926	14.4	11.3	12.9	< 0.001
Smart washing machine	34.0	29.6	< 0.001	29.0	36.0	< 0.001	34.4	25.5	22.1	< 0.001
Smart switch	14.7	13.8	0.200	14.9	14.1	0.187	12.8	10.9	12.9	< 0.001
Smart door lock	17.2	8.9	< 0.001	14.9	15.0	0.818	14.2	8.2	7.0	< 0.001
Smart toilet	13.6	6.7	< 0.001	11.3	12.0	0.234	11.0	9.0	8.1	< 0.001
Smart mosquito repellent	8.7	6.6	< 0.001	8.1	8.2	0.934	6.5	6.4	2.2	< 0.001
Smart clothes hanger	9.8	6.0	< 0.001	7.8	9.6	0.001	8.1	7.4	4.8	0.001
Electric curtain	7.6	5.3	< 0.001	7.6	6.4	0.015	5.2	5.3	5.1	< 0.001
Smart monitoring	11.5	10.5	0.138	11.8	10.7	0.074	9.3	6.9	4.8	< 0.001
**Health smart home**^a^	65.2	47.7	< 0.001	59.5	61.1	0.089	57.7	41.4	33.8	< 0.001
Smart bracelet	30.6	17.5	< 0.001	27.5	26.6	0.274	23.2	12.9	12.5	< 0.001
Body fat scale	31.1	21.5	< 0.001	25.0	31.3	< 0.001	27.2	18.1	15.4	< 0.001
Smart medicine cabinet	5.4	3.8	0.001	5.9	4.2	< 0.001	3.6	4.0	2.6	< 0.001
Smart socket	12.0	11.2	0.220	12.5	11.2	0.031	9.3	10.9	10.3	0.001
Temperature and humidity sensor	7.0	4.6	< 0.001	7.5	5.5	< 0.001	5.7	5.8	1.1	0.001
Danger button	6.1	4.9	0.011	6.6	5.1	0.001	4.2	5.4	6.6	0.001
Smoke transducer	7.7	4.6	< 0.001	7.0	6.7	0.651	5.4	6.4	5.1	0.020
Air purifier	20.0	9.5	< 0.001	17.0	17.2	0.798	16.2	14.3	7.7	< 0.001

^a^ The utilization rate was calculated any use of smart home

## Discussion

In the context of the COVID-19 pandemic, the importance and convenience of digital health are becoming more and more prominent [26, 27], with telemedicine enabling people to have medical consultations at home and avoid infections, and many countries and regions are actively promoting the development of digital health [28, 29]. Identifying the utilization rate of different groups of people for smart home is important to promote the implementation of digital health strategies, and we conducted a cluster analysis of the population based on health skills, divided into three groups: the good health skills group, the middle health skills group, and the poor health skills group, and confirmed the differences in the utilization rate of different groups of people for smart homes through research and analysis.

The good health skills group has the best smart home use of all types of smart homes, both in terms of breadth utilization use and utilization rate. The good health skills group had higher health literacy and chronic disease self-behavior management had a stronger health mindset, were better able to self-manage their diseases [30, 31], and were more likely to use smart home for health management. In addition to this, the population of the good health skills group had the best media use among those, with greater information exposure, and more likely to learn about smart home-related information. In addition to these factors, sociodemographic characteristics also had an impact on smart home use in the three groups.

The age distribution of the good health skills group was younger than the other two groups, with younger people using smart home better than older people, and Alhuwail D's study also indicated that younger people used smart devices more than older people [32]. Our analysis suggests that age affects smart home use in four main ways. Firstly, consumer perceptions are different; while older people show positive attitudes during the experience of VR glasses, they do not have a strong desire to buy them, believing that smart homes are unnecessary in their lives, while younger people associate the experience more with the content being fun [33]. In addition, the China Quality of Life Development Report for the Elderly (2019) shows that about 29.6% of the elderly in China have not attended school, 41.5% have a primary school education, and 25.8% have middle and high school education. It can be seen that the literacy level of older people in China is relatively low [34], and the low level of education limits the use of smart products by older people. Finally, due to the deterioration of physical functions, the elderly has difficulty in understanding the operation of smart home and are worried about not being able to use them independently without help [35, 36].

The good health skills group has the highest proportion of high-income people, and income is one of the influencing factors for smart home use. Smart home products have higher technical requirements for research and development, require a higher level of R&D talents, and have high manufacturing costs. Small companies also find it difficult to enter this industry due to technical and financial problems, making it difficult to achieve scale effects, which leads to the problem of high prices of smart home products, and many users also indicated during the survey that purchase cost is one of the main considerations [37].

The good health skills group had a larger urban population, and place of residence was also a significant influencing factor on smart home use, with urban use better than rural. According to the survey, 84.2% of urban students in the Washington State school district reported being able to use reliable broadband to watch instructional videos, while only 67.5% of rural areas agreed [38]. In China, the Internet gap between urban and rural areas is gradually decreasing, but there is still a 24.1% difference in penetration rates [39]. Smart homes are built on the internet, rural Internet infrastructure is weaker than urban areas, and the digital divide is a barrier to the spread of digital health in rural areas [40, 41], which is one of the reasons for the low utilization rate of smart homes in rural areas. In addition to this, there are differences in access to health information between rural and urban residents, with rural residents having less access to health related information from sources such as specialists, magazines and less frequent use of search engines than urban residents [42], which may contribute to rural residents not knowing enough about digital health and smart home.

Although there are many differences in the smart home uses among different health skills groups, they also show similarities: men have a significantly higher purchase rate for VR glasses, body sensing cars, body bracelets, smart medicine cabinet, smart sockets, and temperature and humidity sensors than women, but women have a significantly higher utilization rate for smart clothes hanger than men. This is mainly determined by lifestyle, income, and subjective willingness. Currently, men generally have higher incomes than the income of women [43, 44], which determines that men have stronger purchasing power for higher-priced smart homes. Secondly, men and women have different household lifestyles; women take on more household chores [45, 46] and maybe more interested in smart clothes hanger related to household chores, while men have more leisure time at home and purchase more entertainment products. In addition, research has shown significant differences in the technology acceptance between men and women, with men showing a stronger intention to use technology [47, 48], while women are more interested in fashion products during consumption [49] and maybe more interested in daily protection of clothing closely related to fashion.

Looking at the three broad smart home categories, smart TVs have the highest utilization rate in the category of entertainment, with VR glasses and body sensing cars having lower utilization rates. This is related to the high frequency of use and usefulness of TVs as traditional homes [50], while VR glasses and body sensing cars may show lower utilization because our sample includes middle aged and elderly people, who are limited by their age and physical mobility [51] to use such exercise products. In the category of functional, smart washing machines and smart air conditioners were used more frequently, while electric curtains, smart clothes hangers, smart mosquito repellents and smart robots were used less frequently. This is related to the influence of the early use of washing machines and air conditioners that are widely used and highly practical, while electric curtains, smart clothes hangers and mosquito extinguishers are less practical and cost-effective, and smart robots are narrowly used, expensive and technically immature, with most of the experiencers expressing a neutral attitude [52]. In the health category, the utilization rate of sports bracelets, body fat scales and air purifiers were high, while the utilization rate of temperature and humidity sensors, hazard buttons, smoke sensors and smart medicine cabinets was low. This is related to the development of supporting facilities such as the Internet, the popularity of sports bracelets, body fat scales and air purifiers, and their low prices. The rest of the products are not very cost effective or practical. And although there are some small manufacturers selling these products, the leading companies are not putting them into production. We could not find any information about these products on the official websites of the larger Chinese smart home manufacturers Xiaomi and Huawei, which may be difficult to trust with consumers.

Implementing a digital health strategy requires a concerted effort by product manufacturers and the government. As older people consume products with greater consideration for ease of use and practicality, manufacturers should pay more attention to the older market and simplify the steps and the interface design of their products. Studies have shown that user involvement in the product design can effectively improve the quality, relevance and prevalence of work [53, 54], and companies can recruit elders as the volunteers to participate in the design process as appropriate to the actual situation. Health products are of importance for aging [55], but the current smart home market has a small range of such products, so manufacturers need to implement the concept of digital health and increase the development, production and promotion of such products. Smart home also can provide better life support to the chronic disease and disabled population, promoting their standard of living and health [55–58]. However, our research shows that the smart home use among the chronic disease and disabled population is poor compared to the healthy population. This may be related to the current types of smart homes, where current manufacturers are designing smart homes more from the perspective of the general population, but the vulnerability of chronic diseases and disabled people and the need for a forte for smart home has led to the fact that current smart home is still lacking in personalized and flexible design for people with disabilities [59], manufacturers should establish good customer relationships with these two types of users, understand their sense of use, pay more in-depth attention to the real device needs of people with disabilities and continuously improve their products. Because of the poor utilization rate of smart home among the elderly, low-income groups, rural residents and women, manufacturers should pay more attention to the positive and negative factors influencing their purchases and broaden the market for their products. The high production costs of companies lead to the high pricing of products, which is one of the major obstacles to people using smart home. The government can enhance policy guidance to attract investors and capital into the smart home market and reduce the production pressure on enterprises. In addition, the government should also encourage enterprises to invest in technology research and development to break through existing technical difficulties, thereby reducing the difficulty of product production and achieving lower product prices. Secondly, because there is also no unified industry standard specification for smart home in China [3], while different companies have different product compatibility [60], leading to the problem that elderly users feel more difficult to use in the process. The relevant authorities should formulate industry standards to address this issue and solve the problem of different product compatibility. At the same time, the government should simultaneously strengthen the network in rural areas to improve rural network coverage and ensure network stability. It is also important to strengthen the promotion of health knowledge in rural areas and to increase rural residents' understanding of high-tech developments in the health field by explaining health knowledge in an easy-to-understand way, such as through posters. Finally, the government should also pay attention to strengthening the health knowledge system of the residents and improving their health skills in general, thereby increasing their demand for using smart homes to manage their health and helping them to better promote it.

Previous studies have confirmed that the use of smart homes can promote health. At the same time, with the World Health Organization and the Chinese government focusing on promoting this technology, it is crucial to explore the factors that influence smart home use. Our study delves into the characteristics of smart home use among different populations, discusses the reasons for the differences between different populations and provides a theoretical basis for the promotion of smart home.

### Strength and limitation

Our study clustered the five influencing factors of health skills: family health, perceived social support, health literacy, chronic disease behavior management and media use into three groups: the good health skills group, the middle health skills group, and the poor health skills group, which contributed to a new way of segmentation. In addition to this, our study confirms that Users with different health skills have differences in smart home use. These findings provide important new insights that have implications for the development of the smart home industry and the implementation of digital health strategies.

Our study has several limitations. Firstly, we conducted survey through respondents retrospectively completing a questionnaire, which may be subject to recall bias. Secondly, our study was a cross-sectional survey and was unable to demonstrate a causal relationship between health skills and smart home use, which could be explored in future studies through longitudinal research. Apart from that, we analyzed health skills through five dimensions: family health, perceived social support, health literacy, chronic disease behavior management, and media use, possibly ignoring the effect of other variables on health skills. Finally, although the 23 smart homes designed into this study were the result of a literature review, expert consultation and a search on Taobao etc., popular shopping sites in China. The final 23 smart homes that were more widely used both domestically and internationally were identified, but in the actual survey, the respondents may also have some smart homes that were not mentioned in our study, which may have cost us a small portion of the data.

## Conclusion

In summary, our study found that smart home use is better among people with good health skills. There are gender differences in the smart home usage categories, with rural populations and older people having worse smart home use. The government and smart home companies need to focus on people with poor smart home use in various ways to promote their utilization rate of smart home for personal health management.

## Supplementary Information

**Additional file 1:** **Table S1.**Variable description. **Table S2.** Perceived social support questions and assignment criteria. **Table S3.** Family health questions and assignment criteria. **Table S4.** Media use and questions and assignment criteria. **Table S5.** Chronic disease self-management study measures questions and assignment criteria. **Table S6.** Health literacy questions and assignment criteria. **TableS7.** Demographic differences of smart home users.

**Additional file 2.**

## Data Availability

The datasets supporting the conclusions of this article are included within the article and its additional files. All raw data can be obtained by sending an email to the corresponding author.

## Abbreviations

HLS: Health Literacy Scale
PSSS: Perceived Social Support Scale
FLS: Family Health Score
Self-management: Chronic disease self-behavioral management
DEXA: X-ray absorptiometry
SHU: Smart Home Use
CDSMS: Chronic Disease Self-management Study Measure
FHS-SF: Family Health Scale-Short Form
HLS-SF12: Short-Form Health Literacy Instrument
