# Supplementary material for: Chinese adult segmentation according to health skills and analysis of their use for smart home: a cross-sectional national survey
Source: BMC Health Serv Res. 2022 Jun 10;22:760. doi: 10.1186/s12913-022-08126-8 (PMC9184334; doi:10.1186/s12913-022-08126-8)
Supplement: Supplementary file 1 — Additional file 1: Table S1.Variable description. Table S2. Perceived social support questions and assignment criteria. Table S3. Family health questions and assignment criteria. Table S4. Media use and questions and assignment criteria. Table S5. Chronic disease self-management study measures questions and assignment criteria. Table S6. Health literacy questions and assignment criteria. TableS7. Demographic differences of smart home users. [file 12913_2022_8126_MOESM1_ESM.docx]

# Supplementary Table S1 Variable Description.

| Variable name | Definition or code |
| --- | --- |
| Age | 1=~18；2=19~45；3=46~59；4=60~75；5=76~90；6=91~ |
| Gender | 1=male; 2=female. |
| Income | Indicates the monthly per capita income of the household  1=0~3000；2=3001~7500；3=~7501 |
| Hukou status | Hukou indicates the respondent’s hukou place and is a special identifier in China. Hukou status affects many aspects of life in China such as buying a house, buying a car, children’s school enrollment and other welfare. 1=urban; 2= rural. |
| Residence | Residence indicates the household living region and is defined by National Bureau of Statistics of the People's Republic of China. 1=urban; 2=rural. |
| Education | 1=Illiteracy; 2= Below secondary school; 3= Secondary Education; 4= College and Bachelor; 5= Master and PhD |
| Public insurance | 0=no; 1=yes |
| Politics | 1=member of the Communist of China, Preparatory member of the Communist Party of China; 2=member of the Communist Youth League; 3=member of the Democratic Revolution, the Democratic League, the Democratic Alliance for the Betterment of Hong Kong, the Democratic Progressive Party, the Agricultural and Labor Party, the Zhi Gong party, the Jiu San Society, the Taiwan Alliance, persons without party affiliation; 4=Crowd |
| Drinking status | Alcohol intake indicates whether the respondent has had an alcoholic beverage in the last 12 months.  0=never had one; 1=drunk, before the last 30 days; 2=drank, within the last 30 days |
| Family type | 1=nuclear families; 2= Conjugal family; 3= Backbone family; 4= Single-parent family; 5=other |
| Location recently | Indicates the place of residence in China within three months.  1=Eastern region; 2=Central region; 3=Western region |
| Marriage | 1=Unmarried；2=Married；3=Divorce；4=Widowed |
| Number of children | 1=zero; 2=one; 3=two; 4=three or more |
| Household | Indicates the number of people living together in the last two months.  0=living alone; 1=one; 2=two; 3=three; 4=four; 5=five or more |
| Chronic disease | Indicates the number of people suffering from chronic diseases。  0=zero; 1=one; 2=two or more |
| Disability | 0=no; 1=yes |
| Work status | 1=in-service; 2=retired; 3=student; 4=no fixed occupation |

# Supplementary Table S2 Perceived Social Support Questions and Assignment Criteria.

There are 12 sentences below, each followed by 7 answers. Please choose one answer after each sentence according to your actual situation.

Extremely disagree=1；Strongly disagree =2；Somewhat disagree=3；Neutral=4

Somewhat agree=5；Strongly agree=6；Extremely agree=7

Total score of 12-36 is low support status, 37-60 is middle support status, 61-84 is high support status

| 1.There are people (relatives, neighbors, colleagues) who are there for me when I have problems |
| --- |
| 2.I can share my joys and sorrows with some people (relatives, neighbors, colleagues) |
| 3.My family is able to solve my problems in a practical way |
| 4.I can get emotional help and support from my family when I need it |
| 5.There are people (relatives, neighbors, colleagues) who are a real source of comfort when I am in trouble |
| 6.My friends can really help me |
| 7.I can rely on my friends in times of difficulty |
| 8.I can talk about my problems with my own family |
| 9.My friends can share my happiness and sadness with me |
| 10.There are people in my life (relatives, neighbors, colleagues) who care about my feelings |
| 11.My family willingly assists me in making decisions |
| 12.I can discuss my problems with my friends |

# Supplementary Table S3 Family Health Questions and Assignment Criteria.

There are 10 sentences below, each followed by 5 answers. Please make your choice in relation to your situation.

Questions 1, 2, 3, 4, 5, 7 and 8 are positively scored: Strongly disagree =1; Somewhat disagree =2; Neither agree nor disagree =3; Somewhat agree =4; Strongly agree =5

Questions 6, 9 and 10 are negative scored: Strongly disagree =5; Somewhat disagree =4; Neither agree nor disagree =3; Somewhat agree =2; Strongly agree =1

The total score is the sum of each question, with a minimum score of 10 and a maximum score of 50, the higher the score, the higher the score, the better the family health index.

| 1.We support each other. |
| --- |
| 2.I feel safe in my family relationships. |
| 3.We help each other in seeking health care services when needed (such as making doctor's appointments). |
| 4.We help each other make healthy changes. |
| 5.We stay hopeful even in difficult times. |
| 6.We do not trust doctors and other health professionals (R). |
| 7.We have people outside of our family we can turn to when we have problems at school or work. |
| 8.If we needed financial help, we have people outside of our family we could turn to for a loan (e.g., for $200) |
| 9.My family did not have enough money at the end of the month after bills were paid (R). |
| 10.My family did not have adequate housing (R). |

# Supplementary Table S4 Media Use.

There are 7 media in the table and 4 answers for frequency of use, please choose the frequency of use for each medium according to the actual situation.

Never use =0; Occasionally use=1; Sometimes use =2; Often use =3; Almost daily= 4

The total score is the sum of each question, with a minimum score of 0 and a maximum score of 28, the higher the score, the better the use of the media.

| 1.Newspaper |
| --- |
| 2.Magazines |
| 3.Radio |
| 4.TV |
| 5.Books (not textbooks) |
| 6.Personal computers (including tablets) |
| 7.Smartphones |

# Supplementary Table S5 Chronic Disease Self-Management Study Measures Questions and Assignment Criteria

There are 6 activities in the table and 5 answers for the time spent on the activity. Please make your choice based on how much time you have actually spent on these activities in the past week.

Not done =0; <30 minutes/week=1; 30-59 minutes/week=2; 1-3 hours/week=3; >3 hours/week=4.

The total score is the sum of each question, with a minimum score of 0 and a maximum score of 20, the higher the score, the better chronic disease self-behavior management.

| 1.Fitness exercises (e.g. cascading movements, dumbbell lifting, etc.) |
| --- |
| 2.Walking |
| 3.Swimming |
| 4.Cycling |
| 5.Exercise with exercise equipment (e.g. treadmill, trampoline, etc.) |
| 6.Other aerobic exercises (e.g. running, table tennis, etc.) |

# Supplementary Table S6 Health Literacy Questions and Assignment Criteria.

There are 12 sentences below, each followed by 4 answers. Please choose one answer after each sentence according to how easy or difficult the following acts are for you.

Very difficult=1; Difficult=2; Easy=3; Very easy=4.

The total score is the sum of each question, with a minimum score of 12 and a maximum score of 48, the higher the score, the higher health literacy.

| 1.Find information on treatments of illnesses that concern you? |
| --- |
| 2.Understand the leaflets that come with your medicine? |
| 3.Judge the advantages and disadvantages of different treatment options? |
| 4.Call an ambulance in an emergency? |
| 5.Find information on how to manage mental health problems like stress or depression? |
| 6.Understand why you need health screenings (such as breast exam, blood sugar test, blood pressure)? |
| 7.Judge which vaccinations you may need? |
| 8.Decide how you can protect yourself from illness based on advice from family and friends? |
| 9.Find out about activities (such as meditation, exercise, walking, Pilates etc.) that are good for your mental well-being? |
| 10.Understand information in the media (such as Internet, newspaper, magazines) on how to get healthier? |
| 11.Judge which everyday behavior (such as drinking and eating habits, exercise etc.) is related to your health? |
| 12.Join a sports club or exercise class if you want to? |

# Supplementary Table S7 Demographic differences of smart home users.

|  | SHU | | Entertainment | | Functional assistance | | Health and safety | |
| --- | --- | --- | --- | --- | --- | --- | --- | --- |
|  | n(%) | P-value | n(%) | P-value | n(%) | P-value | n(%) | P-value |
| **Gender** |  |  |  | 0.303 |  | 0.011 |  | 0.089 |
| Man |  |  | 2639 (46.1%) |  | 3284 (44.8%) |  | 2997 (45.0%) |  |
| Woman |  |  | 3086 (53.9%) |  | 4052 (55.2%) |  | 3667 (55.0%) |  |
| **Age** |  |  |  | <0.001 |  | <0.001 |  | <0.001 |
| ~18 |  |  | 573 (10.0%) |  | 689 (9.4%) |  | 655 (9.8%) |  |
| 19~45 |  |  | 3573 (62.4%) |  | 4567 (62.3%) |  | 4276 (64.2%) |  |
| 46~59 |  |  | 1132 (19.8%) |  | 1460 (19.9%) |  | 1279 (19.2%) |  |
| 60~75 |  |  | 354 (6.2%) |  | 478 (6.5%) |  | 362 (5.4%) |  |
| 76~90 |  |  | 89 (1.6%) |  | 131 (1.8%) |  | 82 (1.2%) |  |
| 91~ |  |  | 4 (0.1%) |  | 11 (0.1%) |  | 10 (0.2%) |  |
| **Income** |  |  |  | <0.001 |  | <0.001 |  | <0.001 |
| ~3000 |  |  | 1510 (26.4%) |  | 1842 (25.1%) |  | 1567 (23.5%) |  |
| 3001~7500 |  |  | 2776 (48.5%) |  | 3631 (49.5%) |  | 3307 (49.6%) |  |
| 7501~ |  |  | 1439 (25.1%) |  | 1863 (25.4%) |  | 1790 (26.9%) |  |
| **Register Residence** |  |  |  | <0.001 |  | <0.001 |  | <0.001 |
| Urban |  |  | 3425 (59.8%) |  | 4433 (60.4%) |  | 4203 (63.1%) |  |
| Rural |  |  | 2300 (40.2%) |  | 2903 (39.6%) |  | 2461 (36.9%) |  |
| **Politics** |  |  |  | <0.001 |  | <0.001 |  | <0.001 |
| CPC |  |  | 1179 (20.6%) |  | 1530 (20.9%) |  | 1473 (22.1%) |  |
| Communist youth league |  |  | 1605 (28.0%) |  | 2045 (27.9%) |  | 1976 (29.7%) |  |
| Other parties |  |  | 2640 (46.1%) |  | 3378 (46.0%) |  | 2903 (43.6%) |  |
| Masses |  |  | 301 (5.3%) |  | 383 (5.2%) |  | 312 (4.7%) |  |
| **Education** |  |  |  | <0.001 |  | <0.001 |  | <0.001 |
| Illiteracy |  |  | 137 (2.4%) |  | 181 (2.5%) |  | 117 (1.8%) |  |
| Below secondary school |  |  | 1051 (18.4%) |  | 1276 (17.4%) |  | 991 (14.9%) |  |
| Secondary Education |  |  | 1033 (18.0%) |  | 1310 (17.9%) |  | 1161 (17.4%) |  |
| College and Bachelor |  |  | 3054 (53.3%) |  | 4011 (54.7%) |  | 3820 (57.3%) |  |
| Master and PhD |  |  | 450 (7.9%) |  | 558 (7.6%) |  | 575 (8.6%) |  |
| **Work status** |  |  |  | <0.001 |  | <0.001 |  | <0.001 |
| In-service |  |  | 2566 (44.8%) |  | 3325 (45.3%) |  | 3108 (46.6%) |  |
| Retired |  |  | 390 (6.8%) |  | 532 (7.3%) |  | 438 (6.6%) |  |
| Student |  |  | 1717 (30.0%) |  | 2133 (29.1%) |  | 2068 (31.0%) |  |
| No fixed occupation |  |  | 1052 (18.4%) |  | 1346 (18.3%) |  | 1050 (15.8%) |  |
| **Family type** |  |  |  | <0.001 |  | 0.004 |  | <0.001 |
| Nuclear family |  |  | 3519 (61.5%) |  | 4408 (60.1%) |  | 4110 (61.7%) |  |
| Conjugal family |  |  | 913 (15.9%) |  | 1197 (16.3%) |  | 1080 (16.2%) |  |
| Backbone family |  |  | 620 (10.8%) |  | 856 (11.7%) |  | 711 (10.7%) |  |
| Single-parent family |  |  | 196 (3.4%) |  | 256 (3.5%) |  | 226 (3.4%) |  |
| Other |  |  | 477 (8.3%) |  | 619 (8.4%) |  | 537 (8.1%) |  |
| **Location recently** |  |  |  | 0.002 |  | 0.620 |  | <0.001 |
| Eastern region |  |  | 2830 (49.4%) |  | 3750 (51.1%) |  | 3474 (52.2%) |  |
| Central region |  |  | 1497 (26.1%) |  | 1896 (25.9%) |  | 1754 (26.3%) |  |
| Western region |  |  | 1398 (24.4%) |  | 1687 (23.0%) |  | 1433 (21.5%) |  |
| **Residence** |  |  |  | <0.001 |  | <0.001 |  | <0.001 |
| Urban |  |  | 4328 (75.6%) |  | 5542 (75.5%) |  | 5222 (78.4%) |  |
| Rural |  |  | 1397 (24.4%) |  | 1794 (24.5%) |  | 1442 (21.6%) |  |
| **Marriage** |  |  |  | <0.001 |  | <0.001 |  | <0.001 |
| Unmarried |  |  | 2323 (40.6%) |  | 2865 (39.1%) |  | 2765 (41.5%) |  |
| Married |  |  | 3210 (56.1%) |  | 4202 (57.3%) |  | 3694 (55.4%) |  |
| Divorce |  |  | 102 (1.8%) |  | 147 (2.0%) |  | 120 (1.8%) |  |
| Widowed |  |  | 90 (1.6%) |  | 122 (1.7%) |  | 85 (1.3%) |  |
| **Children** |  |  |  | <0.001 |  | <0.001 |  | <0.001 |
| No |  |  | 2687 (46.9%) |  | 3380 (46.1%) |  | 3257 (48.9%) |  |
| One |  |  | 1590 (27.8%) |  | 2081 (28.4%) |  | 1961 (29.4%) |  |
| Two |  |  | 1147 (20.0%) |  | 1499 (20.4%) |  | 1196 (17.9%) |  |
| Three or more |  |  | 301 (5.3%) |  | 376 (5.1%) |  | 250 (3.8%) |  |
| **Household** |  |  |  | 0.007 |  | 0.016 |  | 0.013 |
| No |  |  | 546 (9.5%) |  | 736 (10.0%) |  | 668 (10.0%) |  |
| One |  |  | 1795 (31.4%) |  | 2402 (32.8%) |  | 2110 (31.7%) |  |
| Two |  |  | 1661 (29.0%) |  | 2065 (28.2%) |  | 1954 (29.3%) |  |
| Three |  |  | 895 (15.6%) |  | 1138 (15.5%) |  | 1019 (15.3%) |  |
| Four |  |  | 389 (6.8%) |  | 474 (6.5%) |  | 423 (6.4%) |  |
| Five or more |  |  | 433 (7.6%) |  | 514 (7.0%) |  | 485 (7.3%) |  |
| **Public insurance** |  |  |  | <0.001 |  | <0.001 |  | <0.001 |
| No |  |  | 3018(52.7%) |  | 3898(53.1%) |  | 3632(54.5%) |  |
| Yes |  |  | 2707 (47.3%) |  | 3438 (46.9%) |  | 3032 (45.5%) |  |
| **Chronic disease** |  |  |  | 0.020 |  | <0.001 |  | <0.001 |
| No |  |  | 4741 (82.8%) |  | 6078 (82.9%) |  | 5589 (83.9%) |  |
| One |  |  | 661 (11.5%) |  | 839 (11.4%) |  | 734 (11.0%) |  |
| Two or more |  |  | 323(5.7%) |  | 419(5.7%) |  | 341(5.1%) |  |
| **Disability** |  |  |  | 0.247 |  | 0.060 |  | 0.209 |
| No |  |  | 5554 (97.0%) |  | 7119 (97.0%) |  | 6473 (97.1%) |  |
| Yes |  |  | 171(3.0%) |  | 217(3.0%) |  | 191(2.9%) |  |
| **Drinking status** |  |  |  | 0.066 |  | 0.939 |  | <0.001 |
| Within 30 days |  |  | 3354 (58.6%) |  | 4366 (59.5%) |  | 3869 (58.1%) |  |
| Before 30 days |  |  | 694 (12.1%) |  | 872 (11.9%) |  | 816 (12.2%) |  |
| No |  |  | 1677 (29.3%) |  | 2098 (28.6%) |  | 1979 (29.7%) |  |
